# How to Detect Life on Icy Moons

**DOI:** 10.1089/ast.2017.1656

**Published:** 2018-07-01

**Authors:** Mark A. Sephton, Jack Hunter Waite, Tim G. Brockwell

**Affiliations:** ^1^ Impacts and Astromaterials Research Centre, Department of Earth Science and Engineering, Imperial College London, London, United Kingdom.; ^2^ Space Science and Engineering Division, Southwest Research Institute, San Antonio, Texas.

**Keywords:** Europa, Icy moons, Life detection, Mass spectrometry, Organic matter

## Abstract

The icy moons of the outer Solar System present the possibility of subsurface water, habitable conditions, and potential abodes for life. Access to evidence that reveals the presence of life on the icy moons can be facilitated by plumes that eject material from the subsurface out into space. One instrument capable of performing life-search investigations at the icy moons is the MAss SPectrometer for Planetary EXploration/Europa (MASPEX), which constitutes a high-resolution, high-sensitivity multibounce time-of-flight mass spectrometer capable of measuring trace amounts (ppb) of organic compounds. MASPEX has been selected for the NASA Europa Clipper mission and will sample any plumes and the surface-sputtered atmosphere to assess any evidence for habitability and life. MASPEX is capable of similar investigations targeted at other icy moons. Data may be forthcoming from direct sampling but also impact dissociation because of the high speed of some analytes. Impact dissociation is analogous to the dissociation provided by modern analytical pyrolysis methods. Radiolytic dissociation on the europan surface before or during the sputtering process can also induce fragmentation similar to pyrolysis. In this study, we have compiled pyrolysis mass spectrometry data from a variety of biological and nonbiological materials to demonstrate the ability of MASPEX to recognize habitability and detect life in any plumes and atmospheres of icy moons.

## 1. Introduction

Icy moons are a priority for Solar System exploration because of their potential subsurface oceans (Lunine, 2017). Europa (Kivelson *et al.*, 2000), Ganymede (Kivelson *et al.*, 2002), and Callisto (Kivelson *et al.*, 2000) at Jupiter, and Enceladus (Spencer and Nimmo, 2013) at Saturn, all present evidence of subsurface liquid water. Europa has a cratering record that suggests a relatively young icy surface (Smith *et al.*, 1979) that is still mobile (Carr *et al.*, 1998) and a magnetosphere that is consistent with a subsurface liquid brine ocean (Khurana *et al.*, 1998). The cratering record of Enceladus also indicates recent resurfacing (Porco *et al.*, 2006) and plumes can be observed erupting into space (Hansen *et al.*, 2006). Plumes of material venting from Enceladus appear to contain water, methane, carbon dioxide, ammonia, and molecular hydrogen and simple organic compounds (Waite *et al.*, 2017) with recently recognized complex (>200 amu) organic matter (Postberg *et al.*, 2018). The plumes could reflect subsurface hydrothermal activity (Hsu *et al.*, 2015; Waite *et al.*, 2017), a possibility that has been bolstered by geophysical data from the Cassini spacecraft that indicates tidal dissipation in the rocky core (Choblet *et al.*, 2017). Stress fractures on Europa's surface (Greenberg and Geissler, 2002) suggest that venting should also be occurring on Jupiter's smallest Galilean moon and evidence is growing for the presence of europan plumes (Roth *et al.*, 2014; Sparks *et al.*, 2016, 2017), some of which have origins coincident with previously measured hot spots (Spencer *et al.*, 1999).

The NASA Europa Clipper mission aims to orbit Jupiter taking repeated measurements as it passes through Europa's tenuous atmosphere (Phillips and Pappalardo, 2014). The Europa Mission will launch sometime in the first half of the 2020s. There will be 45 flybys of Europa at altitudes varying from 25 to 2700 km over the proposed three-and-a-half-year mission lifetime. In summer 2015, NASA selected nine science instruments for a future mission to Europa. One is the MAss SPectrometer for Planetary EXploration/Europa (MASPEX), a high-resolution, high detection sensitivity multibounce time-of-flight instrument (Brockwell *et al.*, 2016) capable of measuring trace species (ppb for organic compounds) in Europa's atmosphere and in any encountered plumes. MASPEX has the capability to measure species either by direct analysis or at enhanced sensitivities by concentration of analytes in a cryotrap followed by analysis post-flyby (Brockwell *et al.*, 2016).

An exosphere around Europa was discovered over two decades ago (Hall *et al.*, 1995) and is thought to derive from sputtering and radiolysis processes that lead to the ejection of surface materials (Johnson *et al.*, 2009) but may also receive inputs from vented constituents (Roth *et al.*, 2014). Irrespective of the source, the ability of MASPEX to detect organic signals of life in the atmospheres of icy moons can be estimated. The amount of ejected material that can be detected by MASPEX could be as small as 1 kg/s. Sputtering ejects 60 kg/s (Cassidy *et al.*, 2013). These amounts are many times exceeded by the plumes detected by the Hubble Space Telescope, suggesting over 2000 kg/s of outgassed material (Sparks *et al.*, 2017). In the context of MASPEX detecting outgassed material, it should be noted that the Hubble Space Telescope could not see outgassing less than 200–400 kg/s, suggesting that the proposed outgassing rates are minimum values.

MASPEX will measure the composition of gases, ices, and organic compounds and will characterize the habitability of Europa. Analysis of any europan plumes and atmosphere could indicate whether the icy moon harbors conditions suitable for life. Habitability requires liquid water, which is thought to be present, but additional prerequisites are a source of chemical energy to drive metabolism and the presence of organic matter. Organic matter is detectable by MASPEX when it directly enters the mass spectrometry system. In addition, fragmentation can occur following the impact of complex organic matter onto the MASPEX instrument antechamber walls or from radiolytic processes on the surface prior or during the surface sputtering process. If desired, fragmentation on impact with the antechamber can be mitigated by reducing the flyby speed of the spacecraft, relative to the atmosphere, to <5 km/s (<1.3 eV/nucleon), which is generally below the anticipated fragmentation energy limit (Wörgötter *et al.*, 1997). Data from organic fragmentation are complicated but useful owing to the similarity to the fragmentation process that occurs during analytical pyrolysis. If habitability is confirmed, then there is an increase in the probability of life in the icy moon subsurface oceans.

If habitability is confirmed, then seeking evidence of habitation is the next step. Mass spectrometry and pyrolysis mass spectrometry, in the absence of gas chromatographic separation, are established technologies for the rapid detection and recognition of living and fossil organisms (Meuzelaar *et al.*, 1982), including the analysis of microbes (Goodfellow *et al.*, 1997), aquatic biopolymers (Peuravuori and Pihlaja, 1997), land plant biopolymers (Izumi and Kuroda, 1997), and woods from archaeological remains (Lucejko *et al.*, 2009). The advantages of mass spectrometry are commonly enhanced by the addition of a gas chromatography stage where compounds destined for mass spectrometric analysis are separated making the resulting mass spectra simpler. Although direct introduction of analytes to the mass spectrometer is more rapid, the lack of a gas chromatography stage inevitably makes deconvolution of mass spectra more challenging. Yet, approaches are available to mitigate the absence of the gas chromatograph stage.

One method for the improved deconvolution of mass spectra is the use of high-resolution mass spectrometry (Crawford *et al.*, 2017) allowing compounds with the same integer mass (isobars) but different exact mass to be discriminated. For example, high-resolution mass spectrometry has been used to analyze fossil organic assemblages where parental and substituted biphenyls (C_0_ = 154.078, C_1_ = 168.094, C_2_ = 182.109, C_3_ = 196.125, C_4_ = 210.141, C_5_ = 224.156, C_6_ = 238.172) and dibenzofurans (C_0_ = 168.057, C_1_ = 182.073, C_2_ = 196.098, C_3_ = 210.104, C_4_ = 224.120, C_5_ = 238.136) had similar gas chromatography elution characteristics and identical integer masses but could be distinguished by their exact masses (Sephton *et al.*, 1999). As with all complex data sets, statistical methods can also aid in the identification of source materials, including microorganisms (Chun *et al.*, 1993) and other samples of biological interest (Goodacre and Kell, 1996).

Mass spectra data reflect the chemical structure of organic matter. Structure can be used to identify the provenance of organic matter owing to the distinctions between life and nonlife sources (Sephton and Botta, 2005). Nonbiological organic materials are generally characterized by the presence of racemic mixtures, a complete structural diversity, a propensity for branched chain isomers, an exponential decline in amount with increasing carbon number, and the presence of thermally stable aromatic hydrocarbons and polycyclic aromatic hydrocarbons (PAH). Biological organic matter, by contrast, is characterized by relatively specific structures, the presence of chiral preference, the presence of straight chains or cyclic units, no decline in abundance with carbon number, and the absence of thermally stable PAH. Mass spectra of ionized or fragmented organic matter that has been produced by biology can reveal characteristic structurally specific organic entities, while spectra of organic counterparts from nonbiological processes can indicate structurally diverse organic architectures (Sephton and Botta, 2005). MASPEX could, therefore, help to establish biogenicity of any icy moon organic matter.

In life, and in its fossil organic remains in rock samples, organic matter is present as two main solubility classes that can be referred to as low-molecular-weight free compounds and high-molecular-weight macromolecular materials. Free compounds are normally present in proportionally small amounts, while the remaining high-molecular-weight materials represent the dominant fraction. When hosted in mineral matrices on Earth, high-molecular-weight organic matter is called kerogen. If contained within mineral matrices in extraterrestrial objects such as meteorites and asteroids, macromolecular organic materials are commonly called insoluble organic matter.

Organic compounds characteristic of the domains of life (Woese *et al.*, 1990) can be recognized in mass spectra of organic entities found primarily in the free fraction. All known living organisms have lipid membranes and these molecules have additional units attached at their polar ends (*e.g.*, phosphates or sugars). Within membranes are molecular modifiers that help to regulate membrane rigidity. These are isoprenoids, hopanoids, and steroids and they have similar biosynthetic pathways and represent a progressive evolutionary adaptation. The archaea have isoprenoidal structures; bacterial membranes have fatty acid layers with hopanoids; and eukaryotic membranes contain steroids in the place of hopanoids (Killops and Killops, 2005). MASPEX could, therefore, use detection of any biological organic material to help reconstruct potential icy moon ecosystems.

To demonstrate the effectiveness of MASPEX data for habitability assessment and life detection on icy moons, we have used pyrolysis gas chromatography/mass spectrometry data and converted them into pseudodissociation mass spectrometry data. Well understood biogenic and abiogenic material data have been utilized to produce icy moon organic interpretation schemes for habitability and life detection, and statistical methods that can be used on a flyby mission are also outlined. Our icy moon-focused data and methods provide assistance for organic detection missions to the outer Solar System.

## 2. Methods

### 2.1. Samples

A series of organic-rich materials were selected to demonstrate the utility of dissociation mass spectrometry for habitability assessment and life detection on the icy moons. Shales contain long-chain hydrocarbon-dominated materials from microbial or algal inputs and represent lacustrine type I and marine type II organic matter. Coals contain aromatic ring and short-chain hydrocarbon-dominated organic matter from land plants and represent type III organic matter. Reworked and oxidized organic matter assemblages can be derived from any source but contain residual aromatic structures and represent type IV organic matter. A comparison of organic matter types has recognized type IV as the most accurate terrestrial organic analogue for carbonaceous meteorites (Matthewman *et al.*, 2013). Bona fide nonbiological organic matter is found in carbonaceous meteorites.

The sources for our organic matter types were an organic-rich shale from the classic Blue Lias Jurassic Formation in southern England (%C: 0.6), a coal (low-volatile bituminous) from the Lorraine Seam at Siege Reumaux, France (%C: ca. 80), charcoalified wood from the Cretaceous Wealden beds of southern England (%C: 53.5), and a sample of reworked organic matter from a paleosol bed in the Jurassic-Cretaceous Purbeck Limestone Group in southern England, United Kingdom (%C: 3.5). Carbonaceous meteorites were carbonaceous chondrites of low petrologic types (Pearson *et al.*, 2006), namely Orgueil (CI1; %C: 4.9), Murchison (CM2; %C: 2.7), and Allende (CV3; %C: 0.3).

### 2.2. Data acquisition and conversion

When mounted on the Europa Clipper spacecraft, the MASPEX instrument will be traveling at <5 km/s and organic matter detected in ice/dust grains will contain both intact organic compounds encountered at lower combined speeds and organic fragments generated by impacts on the chamber walls when encountered at higher combined speeds (Jaramillo-Botero *et al.*, 2012). The MASPEX direct detection and impact fragmentation processes will be analogous to those observed during thermal evaporation and thermal dissociation by analytical pyrolysis, respectively. We extracted selected examples from a pre-existing pyrolysis gas chromatography/mass spectrometry data set and converted these data to pyrolysis mass spectrometry equivalents by summing the total ion chromatographs for each sample to produce a single spectrum. We inferred that the “pseudo” pyrolysis mass spectrometry data could represent an analogue to that obtained by MASPEX mass spectrometry and impact dissociation mass spectrometry. The integer mass data were generated from the organic-rich samples described above; any loss in diagnostic capability from less organic-rich samples at the icy moons may be partly or completely offset by the high-resolution accurate mass capabilities of MASPEX (Brockwell *et al.*, 2016).

The original pyrolysis gas chromatography/mass spectrometry measurements involved the heating of organic matter at 20,000°C/s followed by introduction of the liberated organic compounds onto a gas chromatograph. During the thermal experiments, low temperatures (thermal extraction) simply evaporated the free organic compounds while higher temperatures (pyrolysis) also thermally dissociated any high-molecular-weight components. All experiments reported in this article were performed at pyrolysis temperatures. The gas chromatograph was fitted with a capillary column that separates compounds before their introduction to a mass selective detector, which generated electron impact fragmentation patterns. The complete pyrolysis gas chromatography/mass spectrometry data set was converted to pyrolysis mass spectrometry data by summing the responses from elution times of 5 to 45 min in a total ion current to produce a single spectrum. Because the objective of this article is to explore life detection methods, a scan range of 40 to 250 amu was used to enhance the diagnostic organic responses that may be encountered by MASPEX.

## 3. Results

### 3.1. Mass spectra

The contributions to the mass spectra were identified by recognizing patterns of responses that correspond to fragmentation patterns of particular compound classes. The fragmentation behavior of organic structures is a characteristic feature. Many organic assemblages, and particularly those represented by fragmented high-molecular-weight macromolecular materials, are dominated by specific types of structure. For example, the organic fragmentation products of meteorites are dominated by products from aromatic structures, algae by fragments of aliphatic biopolymers, and woody land plants by products from phenolic biopolymers. Characteristic ions found within mass spectra of common natural organic materials are presented in Table 1.

**Table 1.** Common Biological (Fossil) and Nonbiological Organic Compounds and Their Characteristic Fragment Ions Expressed as Integers

	*Compound type*	*Fragment ions (*m/z*)*	*Comment*
Chains and rings			
Straight chains	Alkanoic acids	60, 73, 87, 101, etc.	Important membrane lipids in bacteria and eukarya. Energy store compounds. Specificity of longer chains (fatty acids) commonly reflects biosynthesis.
	Alkanes	29, 43, 57, 71, 85, 99, 113, 127, 141, etc.	Decarboxylation products of fatty acids. Thermal product of aliphatic biopolymers. Specificity of longer chains commonly reflects biosynthesis.
	Alkenes	27, 41, 55, 69, 83, 97, 111, 125, 139, etc.	Characteristic products of rapid (analytical) fragmentation of aliphatic polymer. Specificity of longer chains reflects biosynthesis.
Structurally highly specific	Isoprenoidal alkanes	29, 43, 57, 71, 85, 99, 113, 127, 141, etc.	Important membrane lipids in archaea. Elevated ion abundance at branch point (*e.g.*, *m/z* 183) makes them distinct from straight-chain alkanes. Regular branching pattern reflects biosynthesis.
	Terpenoids	191, 217	Important membrane lipids for bacteria (hopanoids, *m/z* 191) and eukarya (steroids, *m/z* 217). Complex structure indicates biosynthetic process.
Heteroatomic			
Oxygen containing	Polyols (*e.g.*, glycogen)	*m/z* 32, 43, 72, 74, 84, 96, 98, 102, 110, 112, etc.	Sugar-related compounds. Important energy stores for all organisms.
Nitrogen containing	Amines	30, 44, 58, 72, 86, 100, etc.	Amines make up part of the structure of amino acids. Essential components for proteins and enzymes.
Sulfur containing	Alkylthiophenes	45, 57, 58, 69, 71, 83–85, 97, 98, 109, 110, 111, 112, etc.	Diagenesis adds sulfur to any double bonds present.
Aromatic	Alkylbenzenes	78, 91, 92, 105, 106, 119, 120, 133, 134, etc.	Aromatic compounds are common products of thermal metamorphism of pre-existing organic matter. Aromatic compounds are also common in meteorites.
	Alkylnaphthalenes	128, 141, 142, 155, 156, etc.	
	Alkylbenzothiophenes	134, 147, 148, 161, 162, etc.	
	Alkylphenols	77, 91, 94, 107, 108, 121, 122, etc.	
	Polycyclic aromatic hydrocarbons	128, 152, 154, 166, 178, 202, 228, 252, 276, 278, etc.	PAH are not synthesized by biology. They are found in meteorites, partial combustion products, and metamorphosed organic matter.

PAH = polycyclic aromatic hydrocarbons.

### 3.2. Organic-rich shales

**FIG. 1.**
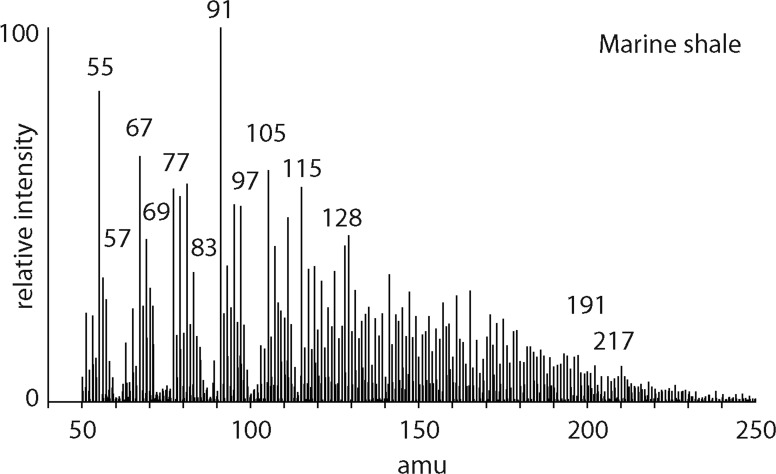
Mass spectrum of the products from pyrolysis of Blue Lias marine shale at 600°C.

The pyrolysis mass spectrometry response for shales (Fig. 1) is characterized by an abundance of peaks corresponding to the alkene series where fragmentation produces units that are different in mass by 14 amu (*m/z* 55, *m/z* 69, *m/z* 83, *m/z* 97, etc.). The alkane series is evident where fragmentation produces units that are different in mass by 14 amu (*m/z* 57, *m/z* 71, *m/z* 85, etc.). Peaks corresponding to benzenes (C_0_ *m/z* 77/78, C_1_ *m/z* 91/92, C_2_ *m/z* 105/106) and naphthalenes (C_0_ *m/z* 128) are present. Hopanoids (*m/z* 191) and steroids (*m/z*/217) are also evident.

### 3.3. Coals

**FIG. 2.**
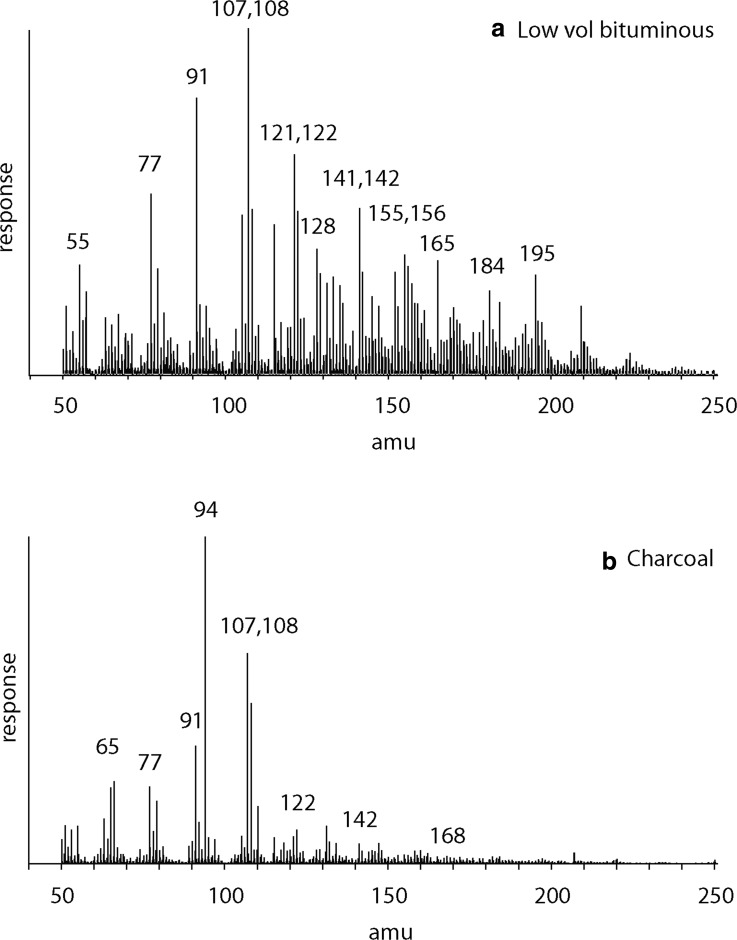
Mass spectrum of the products from pyrolysis of **(a)** low-volatile bituminous coal and **(b)** charcoalified wood at 600°C.

The pyrolysis mass spectrometry responses for coals (Fig. 2) are characterized by an abundance of peaks corresponding to benzenes (C_0_ *m/z* 77/78, C_1_ *m/z* 91/92, C_2_ *m/z* 105/106, C_3_ *m/z* 119/120, C_3_ *m/z* 134), phenols (C_0_ *m/z* 94, C_1_ *m/z* 107/108, C_2_ *m/z* 121/122), naphthalenes (C_0_ *m/z* 128, C_1_ *m/z* 141/142, C_2_ *m/z* 155/156, C_3_ *m/z* 169/170, C_4_ *m/z* 183/184), dibenzofuran (*m/z* 168), and anthracene/phenanthrene (*m/z* 178). There are differences between the responses for the low-volatile bituminous coal and charcoalified wood. The low-volatile bituminous coal contains a wide range of responses reflecting substituted benzenes, naphthalenes, and phenols (Fig. 2a), while the charcoal contains a more limited range of responses dominated by phenols (Fig. 2b).

### 3.4. Reworked organic matter

**FIG. 3.**
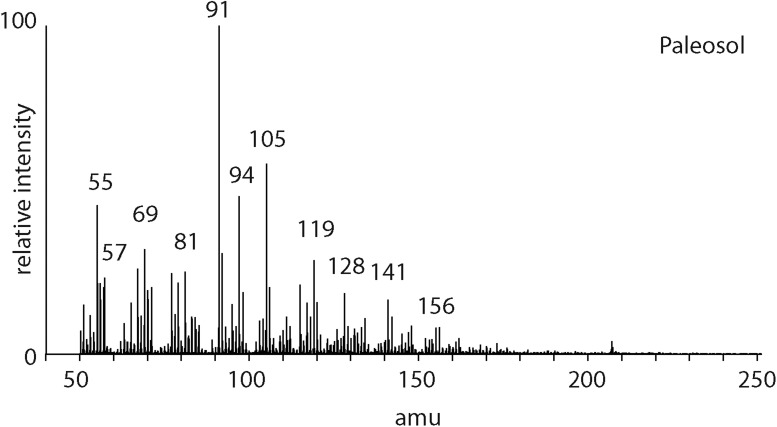
Mass spectrum of the products from pyrolysis of a fossil soil at 600°C.

The pyrolysis mass spectrometry response for reworked organic matter in paleosols (Fig. 3) is characterized by an abundance of peaks corresponding to benzenes (C_0_ *m/z* 77/78, C_1_ *m/z* 91/92, C_2_ *m/z* 105/106), naphthalenes (C_0_ *m/z* 128, C_1_ *m/z* 141/142, C_2_ *m/z* 155/156), and phenol (C_0_ *m/z* 94). The alkene/alkane series is evident where fragmentation produces units that are different in mass by 14 amu (*m/z* 55 + 57, *m/z* 69 + 71, etc.).

### 3.5. Carbonaceous meteorites

**FIG. 4.**
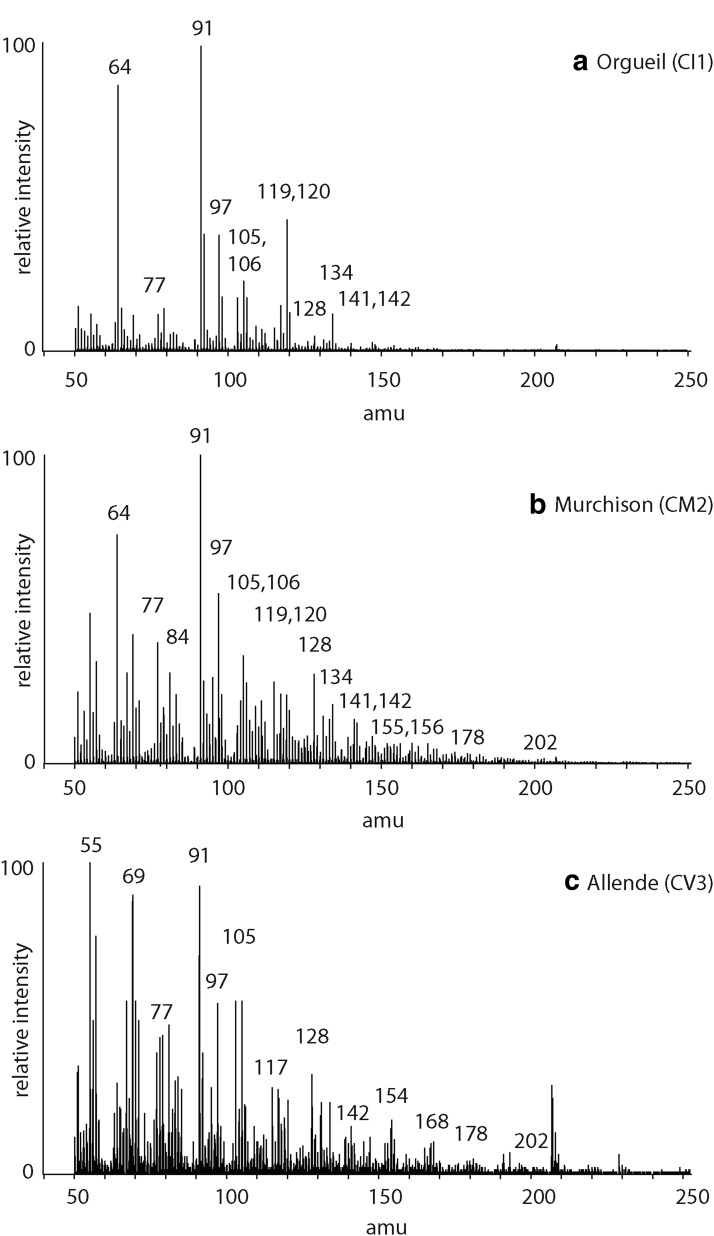
Mass spectrum of the products from pyrolysis of the **(a)** Orgueil (CI1), **(b)** Murchison (CM2), and **(c)** Allende (CV3) carbonaceous meteorites at 650°C.

The pyrolysis mass spectrometry response for nonbiological organic matter in carbonaceous meteorites (Fig. 4) is characterized by an abundance of peaks corresponding to benzenes (C_0_ *m/z* 77/78, C_1_ *m/z* 91/92, C_2_ *m/z* 105/106) and naphthalenes (C_0_ *m/z* 128, C_1_ *m/z* 141/142, C_2_ *m/z* 155/156). Sulfur (*m/z* 64) and thiophenes (C_0_ *m/z* 84, C_1_ *m/z* 97) are present. Alkenes and alkanes are absent in Orgueil and Murchison, but the *m/z* 55, 69, and so on alkene responses appear in the data for Allende. There are differences in the responses for the three meteorites. The lower petrologic-type Orgueil and Murchison meteorites produce more prominent responses from *m/z* 91 (methyl benzene) and *m/z* 105 (dimethyl benzene), while the higher petrologic-type Allende meteorite generates more obvious responses from *m/z* 128 (naphthalene) and *m/z* 154 (biphenyl).

## 4. Discussion

### 4.1. Mass spectra and life detection

The distinctiveness of biological organic matter derives from the specific biochemical needs of organisms catered for by enzymatically selective synthesis. Nonbiological organic synthesis, by contrast, most often displays little preference and the products are randomly generated. For nonbiological organic matter, more carbon atoms mean more potential isomers and the growth in numbers of isomers increases in an exponential manner. For instance, 12 carbon alkanes have more than 350 isomers, whereas 20 carbon alkanes have more than 350,000 isomers. Mass spectra from ultimately biological organic matter (Figs. 1–3) reflect structurally specific architectures, while the mass spectra for nonbiological organic matter reflect greater structural diversity (Fig. 4). The presence of complex organic compounds reflective of specific domains of life can be recognized in mass spectra by their characteristic ions, which are *m/z* 183 for isoprenoids, *m/z* 191 for hopanoids, and *m/z* 217 for steroids. Mass spectra for the organic-rich shale (Fig. 1) display clear responses for hopanoids (*m/z* 191) and steroids (*m/z* 217) indicating contributions to the organic inventory from bacteria and eukarya, respectively.

### 4.2. Free and macromolecular organic materials

During thermal dissociation experiments, low temperatures simply evaporate the free organic compounds, while higher temperatures also thermally dissociate the high-molecular-weight components. An example of evidence of free and macromolecular organic fractions in mass spectra is provided by the shale data (Fig. 1) where hopanoids and steroids (*m/z* 191 + 217) from the free fraction are present alongside alkene/alkane responses (*m/z* 55 + 57) produced by the degradation of a high-molecular-weight aliphatic network. Greater levels of thermal energy progressively increase the proportions of macromolecular fragments present in the thermal extract. Because heat, electron impact, and collision all increase the internal energy of an organic compound, the respective fragmentation processes can be expected to share common mechanisms (Vékey, 1996). A similar interpretation, therefore, can be applied to impact dissociation of organic matter (Wörgötter *et al.*, 1997; Rakov *et al.*, 2002; Delcorte *et al.*, 2009) from icy moons in the outer Solar System. Minimum fragmentation of organic compounds occurs at <5 km/s flyby speeds, while greater impact energies would be expected to lead to higher proportions of macromolecular fragments present in the mass spectra (Jaramillo-Botero *et al.*, 2012).

### 4.3. Mass spectra and straight chain aliphatic hydrocarbons

In the mass spectra of samples with an ultimately biological origin, there are often straight chain hydrocarbons in the free fractions. Free saturated hydrocarbons are highlighted by the *m/z* 57 response and can originate from fatty acids that are decarboxylated following burial. Hydrocarbon series can be recognized in our data in the organic-rich shales and paleosols (Figs. 1 and 3). The presence of unsaturated and saturated hydrocarbons together can indicate fragmentation of a high-molecular-weight, macromolecular organic structure dominated by hydrocarbon units. The location of fragmentation leads to the production of a terminal alkene and an alkane. In the absence of additional hydrogen, the R–CH_2_–H_2_C–R must transform to R–CH_3_ and R CH_2_. A series of normal alkenes and normal alkanes that range to high molecular weights therefore indicate the presence of a structurally specific polymer. Such polymers are produced by biology for protective roles in the parent organisms and, for example, have been recognized in algae (algaenan) and plant leaves (cutan) (DeLeeuw and Largeau, 1993). The presence of a homologous series of alkenes and alkanes is a biosignature that can be readily identified in mass spectra data.

### 4.4. Mass spectra and isomers of aromatic hydrocarbons

Substituted benzenes can be recognized in both the free and macromolecular fractions of coals, paleosols, and carbonaceous meteorites (Figs. 2–4). The dominant dimethylbenzene and trimethylbenzene isomers can be recognized by their characteristic relative abundance of ions (Table 2).

**Table 2.** Substituted Benzenes Listed in Their Elution Order on a Nonpolar Chromatography Column Alongside Molecular Weights and Characteristic Ions with Percentages in Parentheses (Sephton, 2013) and After (Hartgers *et al.*, 1994)

*Compound*	*Molecular weight*	*Characteristic ions (%)*
Ethylbenzene	106	91 (44), 105 (3), 106 (14)
1,3-dimethylbenzene (meta-)	106	91 (32), 105 (9), 106 (21)
1,4-dimethylbenzene (para-)	106	91 (33), 105 (10), 106 (21)
1,2-dimethylbenzene (ortho-)	106	91 (35), 105 (6), 106 (14)
Styrene	104	78 (12), 103 (15), 104 (36)
Isopropylbenzene	120	91 (3), 105 (49), 120 (13)
n-Propylbenzene	120	91 (65), 105 (2), 120 (13)
1-methyl-3-ethylbenzene	120	91 (5), 105 (43), 120 (14)
1-methyl-4-ethylbenzene	120	91 (5), 105 (46), 120 (13)
1,3,5-trimethylbenzene	120	105 (35), 119 (5), 120 (21)
1-methyl-2-ethylbenzene	120	91 (5), 105 (46), 120 (14)
1,2,4-trimethylbenzene	120	105 (38), 119 (5), 120 (19)
1,2,3-trimethylbenzene	120	105 (40), 119 (5), 120 (19)

The ratios of meta-, para-, and ortho-dimethylbenzenes are, to some extent, diagnostic for the provenance of these organic units (Table 3). The (meta+para)/ortho or (1,3-dimethylbenzene +1,4-dimethylbenzene)/1,2-dimethylbenzene ratio can be used as a parameter for comparisons. Meta-isomers are more thermodynamically stable than other forms (Everdell, 1967) and are therefore preferred at higher temperatures. Because many environments where aromatic compounds are synthesized have specific temperatures, their contributions to organic matter can be recognized. Similar arguments can be applied for trimethylbenzenes.

**Table 3.** Relative Abundances or Responses of Dimethylbenzene Isomers Associated with Various Formation and Modification Mechanisms

*Mechanism*	*Environment*	*Meta-*	*Para-*	*Ortho-*	*m+p/o*
Space and laboratory processes					
High temperature (500°C)	Stellar atmosphere	5	3	1	8
High temperature (800°C)	Stellar atmosphere	4	2	2	3
Ion-molecule reaction	Molecular cloud	6	2	2	4
Radiation resistance	Grain mantle	High	Med	Low	High
Fischer–Tropsch	Solar nebula	10.4		2.8	3.7
Degradation resistance	Parent body	Med	High	Low	Med
Catalytic cyclization	Earth laboratory	27	7	33	1
Terrestrial biosphere					
Blue Lias (type I kerogen)	Earth biosphere	2		1	2
Low-volatile bituminous coal (type III kerogen)	Earth biosphere	27		10	2.7
Charcoal (type IV kerogen)	Earth biosphere	19		5	3.8

Space and laboratory data from Sephton (2013) and references therein; biological data from samples used in this study.

On Earth, the thermodynamically unfavorable structures are selected by enzymatic processes that prioritize the biochemical utility of organic arrangements. For example, para-dimethylbenzenes in coals reflect relics of lignin biopolymer chemistry (Hartgers *et al.*, 1994) and lead to the elevated (meta+para)/ortho-dimethylbenzene ratios, especially in coal and charcoal samples that retain indicators of their biochemical heritage (Table 3). Phenols also represent residual units from land plant lignin (Hartgers *et al.*, 1994) and are, therefore, evident in coals and charcoals (Fig. 2).

The isomers of tetramethylbenzenes provide further useful opportunities for source recognition. The presence of four substitutions on the benzene unit gives the opportunity for substantial isomeric diversity, which, in a nonbiological system, leads to carbon being distributed relatively homogeneously. Biological enzymatically driven synthesis, however, would be highly specific and particular substituted benzene isomers can be diagnostic for biological compounds. The 1,2,3,4-tetramethylbenzene is an example of a highly specific structure inherited from the carotenoid pigment biological precursor (Summons and Powell, 1987). The 1,2,3,4- form can be readily distinguished from other isomers in mass spectra by the dominance of the *m/z* 134 rather than the usual *m/z* 133 displayed by the other tetramethylbenzene isomers.

### 4.5. Mass spectra and alteration histories

**FIG. 5.**
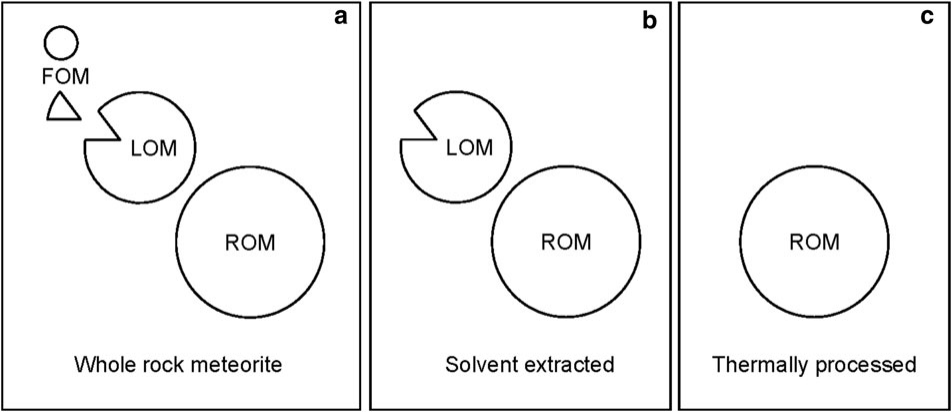
The different types of organic matter in carbonaceous meteorites. Each type of organic matter responds differently to pyrolysis and the relative abundances of the three fractions can reflect preterrestrial aqueous and thermal processing, after (Sephton *et al.*, 2003). FOM, free organic matter; LOM, labile organic matter; ROM, refractory organic matter. **(a)** all fractions are present in the whole rock meteorite, **(b)** FOM can be removed by solvent extraction, **(c)** LOM can be partly or completely removed by aqueous and/or thermal processing.

Secondary processing by heat and/or water can leave a record in organic matter. For instance, there are differences in the mass spectra responses for the three meteorites that can be related to their preterrestrial histories (Fig. 4). The carbonaceous chondrites contain free and macromolecular organic matter that can be further subdivided into three operational types (Fig. 5) (Sephton *et al.*, 2003). Free organic matter is soluble in organic solvents. Labile organic matter has a macromolecular structure making it insoluble in solvents but can be released readily by laboratory pyrolysis methods to reveal small substituted aromatic units with occasional heteroatoms. Refractory organic matter is unaffected by solvents and is relatively resistant to pyrolysis methods and reveals larger substituted and parental PAH units. In whole rock meteorites, the macromolecular entities (labile and refractory organic matter) are by far the most abundant organic components present. The relative abundance of labile to refractory organic matter is a reflection of the degree of secondary processing by water and heat on the meteorite parent body (Sephton *et al.*, 2003, 2004). The large response from *m/z* 91 (methyl benzene) and 105 (dimethyl benzene) in Orgueil and Murchison (Fig. 4a, b) reflects aqueously altered meteorites that have retained labile organic matter, while the elevated *m/z* 128 (naphthalene) and 154 (biphenyl) in Allende (Fig. 4c) reflect a more thermally altered meteorite dominated by more refractory organic matter.

### 4.6. Mass spectra and statistical analysis

Statistical methods (Bayes, 1763) can benefit organic detection activities on icy moons. For statistical methods to be applied, the ability of the instrument or method to detect true positives and true negatives must be known. The true positive rate (also known as sensitivity) is the probability that a test result will be positive when the target analyte (*e.g.*, indigenous organic matter) is present. The true positive rate is calculated by taking the number of true positives and dividing by the total number of samples that contain the target analyte, that is, true positive rate = true positives/(true positives + false negatives). The true negative rate (also known as specificity) is the probability that a test result will be negative when the target analyte is not present. The true negative rate is calculated by taking the number of true negatives and dividing by the total number of samples that do not contain the target analyte, that is, true negative rate = true negatives/(true negatives + false positives).

The ability of an instrument or method to produce true and false positives or true and false negatives can be determined by first choosing a material that is known to be barren of the target analyte and therefore should produce a true negative result, and second, adding known quantities of well-characterized target analyte to produce a mixture that should generate a true positive. These “gold standard” samples can then be used to perform a series of analyses by the instrument intended to make the measurement and the true and false positive plus true and false negative data sets (Gordon and Sephton, 2016).

True positive rate and true negative rate data sets can be used to calculate likelihood ratios for organic detection instruments or methods. A likelihood ratio expresses the likelihood of an expected response for a sample containing the target analyte compared with the likelihood of an expected response for a sample without the target analyte. Values for likelihood ratios extend from zero to infinity. The positive likelihood ratio (the likelihood of the correct response for a sample containing the target analyte) is calculated as follows: positive likelihood ratio = true positive rate/(1 − true negative rate), while the negative likelihood ratio (the likelihood of the correct response for a sample that does not contain the target analyte) is calculated thus: negative likelihood ratio = (1 − true positive rate)/true negative rate. Positive likelihood ratios attract the most attention and are the focus of the following discussion on achieving greater degrees of certainty for detection of indigenous organic matter on icy moons.

A premeasurement probability is needed to express how likely the target analyte is to be present in the expected samples. The premeasurement probability can be obtained in two ways. If no previous campaign exists, or if previous data are not appropriate, the premeasurement probabilities can be obtained by using a number of analog materials. Icy moon analogues include hydrothermal vent settings and soda lakes (Glein *et al.*, 2015). For example, if using a battery of techniques it is found that 5% of icy moon analogues' soda lake deposits contain the target analyte (*e.g.*, indigenous organic matter), then the premeasurement probability of similar materials in the plumes or atmospheres of icy moon containing the same material becomes 0.05. Alternatively, if a relevant previous sampling campaign exists, then the fraction of samples found to contain the target analyte can be used as the probability.

For the purpose of demonstration below, the premeasurement probability of organic detection in the plumes and atmospheres of icy moons can be derived from the Cassini mission data. The Cassini Cosmic Dust Analyzer performed measurements in the plumes of Enceladus, and the E ring around Saturn they supply, and determined that 25% of total particles contained organic compounds (Postberg *et al.*, 2008) and 3% contained complex >200 amu organic matter (Postberg *et al.*, 2018). Table 4 summarizes the steps that are taken to assess the effect of a positive measurement of (1) indigenous organic compounds and (2) complex >200 amu organic matter. First the premeasurement probability is converted to odds as follows: odds = probability/(1 – probability). The postmeasurement odds are then calculated by multiplication with the positive likelihood ratio; thus, postmeasurement odds = premeasurement odds × likelihood ratio. The new postmeasurement odds can then be converted to a probability as follows: probability = odds/(1 + odds).

**Table 4.** Probability Calculations for Organic Detection in Icy Moon Samples with Varying True Positive Rates and True Negative Rates and Different Associated Positive Likelihood Ratios

	*← Method with increasing TPR, TNR, and LHRs*					
Organic compounds						
True positive rate = true positives/(true positives + false negatives)	0.99	0.95	0.90	0.75	0.60	0.50
True negative rate = true negatives/(true negatives + false positives)	0.99	0.95	0.90	0.75	0.60	0.50
Positive likelihood ratio = true positive rate/(1 − true negative rate)	99.0	19.00	9.00	3.00	1.50	1.00
Premeasurement probability (fraction of particles with the analyte)	0.25	0.25	0.25	0.25	0.25	0.25
Premeasurement odds = probability/(1 – probability)	0.33	0.33	0.33	0.33	0.33	0.33
Postmeasurement odds = premeasurement odds × likelihood ratio	33.0	6.33	3.00	1.00	0.50	0.33
Postmeasurement probability = odds/(1 + odds)	0.97	0.86	0.75	0.50	0.33	0.25
Postmeasurement probability (%)	97	86	75	50	33	25
Complex >200 amu organic matter						
True positive rate = true positives/(true positives + false negatives)	0.99	0.95	0.90	0.75	0.60	0.50
True negative rate = true negatives/(true negatives + false positives)	0.99	0.95	0.90	0.75	0.60	0.50
Positive likelihood ratio = true positive rate/(1 − true negative rate)	99.0	19.0	9.00	3.00	1.50	1.00
Premeasurement probability (fraction of particles with the analyte)	0.03	0.03	0.03	0.03	0.03	0.03
Premeasurement odds = probability/(1 − probability)	0.03	0.03	0.03	0.03	0.03	0.03
Postmeasurement odds = premeasurement odds × likelihood ratio	3.06	0.59	0.28	0.09	0.05	0.03
Postmeasurement probability = odds/(1 + odds)	0.75	0.37	0.22	0.08	0.04	0.03
Postmeasurement probability (%)	75	37	22	8	4	3

Single measurements are likely in the icy moon environment; so very high positive likelihood ratios will provide the best certainty of detection.

LHR = likelihood ratio; TNR = true negative ratio; TPR = true positive ratio.

In general, greater statistical certainty is obtained by measuring a sample with the highest premeasurement probability, using an instrument with a high likelihood ratio, and making multiple positive detections. During an icy moon flyby, certain constraints will be encountered: (1) the pre-measurement probability of the particles is fixed by experiences on the previous mission and (2) there are unlikely to be multiple opportunities to measure the same sample. Consequently, the most decisive measurements must be obtained by using an instrument or method with the highest possible positive likelihood ratio. If the positive likelihood ratio for the instrument or measurement is above 1, then once an appropriate measurement has been made that provides evidence for the detection of a given analyte, and then, the possible presence of that analyte obtains a higher probability. The higher the positive likelihood ratio, the greater the increase in probability following a positive detection.

The example in Table 4 indicates that the certainty of a positive detection increases when a positive result is obtained. With a low positive likelihood ratio of 3, a single positive detection would raise the probability of analyte presence from 25% to 50% for organic compounds and 3% to 8% for complex >200 amu organic matter. With a high positive likelihood ratio of 99, a single positive detection would raise the probability of analyte presence from 25% to 97% for organic compounds and 3% to 75% for complex >200 amu organic matter. Table 4 clearly reveals that an instrument or method with a higher likelihood ratio is more effective at increasing the probability of the presence of an analyte once evidence is provided by means of a positive measurement response, with perfect (∼1) true positive rates and true negative rates, and associated likelihood ratios, resulting in practically unequivocal data.

The results in Table 4 indicate that the probabilities of the icy moon samples containing the target analyte increase following a single positive detection. The calculation outlined above specifies indigenous organic compounds and complex >200 amu organic matter as the target analytes. If the target analytes were modified to be indigenous organic matter that indicates the presence of life, then the premeasurement probabilities and the true positive rate and the true negative rate are simply modified to reflect this more exacting type of desired evidence.

Suggestions, therefore, for future organic matter detection missions and instruments to icy moons are as follows:

(1) On Earth, instruments should be tested with samples of known organic matter content to determine their positive likelihood ratios.
(2) When sampling icy moons, greatest confidence is derived from positive detections on samples with the highest premeasurement probability of the target analyte (*e.g.*, indigenous organic compounds or complex organic matter) being present based on previous sampling campaigns or Earth analog studies.
(3) On the mission, for an instrument with positive likelihood ratios above 1, a positive detection of organic carbon will result in an increase in the premeasurement probability of indigenous organic carbon being present.
(4) For a single or fixed number of analyses, the highest positive likelihood ratio method or instrument will provide the greatest probability that indigenous organic matter is present.

The type of test used is instrument, analyte, and parameter specific. Data such as that presented in this article can be used to design tests of varying sensitivity and specificity. The tests can be particular to just life itself (*e.g.*, an organic compound displaying a level of complexity that could not be produced by an abiotic process) or a specific type of life (*e.g.*, an organic compound that reflects a particular biochemical function needed by an organism adapted to a particular environment). Individual organic compounds or combinations of organic compounds can constitute diagnostic parameters (*e.g.*, Peters *et al.*, 2005). Analog testing campaigns with instruments similar to flight hardware and samples of known content can provide the required data for the statistical analyses.

## 5. Conclusions

Mass spectra derived from the MASPEX instrument for the Europa Clipper mission could detect any organic matter present in the plumes of Europa and will characterize its structure. MASPEX is also ideally suited to similar investigations on other icy moons in the outer Solar System such as Ganymede, Callisto, and Enceladus. Laboratory investigations of pyrolysis mass spectrometry data, which can be considered pseudodissociation mass spectrometry data analogous to that which will be obtained by MASPEX, indicate the ability of this type of analysis to detect organic matter and distinguish between nonbiological and biological materials. By examining indicators of the dominant isomers present, the data also have the potential to reveal the environment in which the organic matter was generated. Characteristic ions in the mass spectra could potentially indicate specific domains of life and therefore evolutionary development and ecosystem diversity. Statistical considerations suggest preparation for icy moon missions should include the analysis of standard and analog materials. Instruments and methods should be developed with the highest possible likelihood ratios.

## Abbreviations Used

MASPEX: MAss SPectrometer for Planetary EXploration/Europa
PAH: polycyclic aromatic hydrocarbons

## References

[B1] Bayes T. (1763) An essay towards solving a problem in the doctrine of chances. Philos Trans R Soc Lond 53:370–418 1857193

[B2] Brockwell T.G., Meech K.J., Pickens K., Waite J.H., Miller G., Roberts J., Lunine J.I., and Wilson P. (2016) The mass spectrometer for planetary exploration (MASPEX). In *Aerospace Conference 2016 IEEE*, IEEE, Big Sky, MT

[B3] Carr M.H., Belton M.J.S., Chapman C.R., Davies M.E., Geissler P., Greenberg R., McEwen A.S., Tufts B.R., Greeley R., Sullivan R., Head J.W., Pappalardo R.T., Klaasen K.P., Johnson T.V., Kaufman J., Senske D., Moore J., Neukum G., Schubert G., Burns J.A., Thomas P., and Veverka J. (1998) Evidence for a subsurface ocean on Europa. Nature 391:363–365 9450749 10.1038/34857

[B4] Cassidy T.A., Paranicas C.P., Shirley J.H., Dalton J.B., Teolis B.D., Johnson R.E., Kamp L., and Hendrix A.R. (2013) Magnetospheric ion sputtering and water ice grain size at Europa. Planet Space Sci 77:64–73

[B5] Choblet G., Tobie G., Sotin C., Běhounková M., Ccaron;adek O., Postberg F., and Souček O. (2017) Powering prolonged hydrothermal activity inside Enceladus. Nat Astron 1:841–847

[B6] Chun J., Atalan E., Ward A.C., and Goodfellow M. (1993) Artificial neural network analysis of pyrolysis mass spectrometric data in the identification of Streptomyces strains. FEMS Microbiol Lett 107:321–326 8472913 10.1111/j.1574-6968.1993.tb06051.x

[B7] Crawford E.A., Gerbig S., Spengler B., and Volmer D.A. (2017) Rapid fingerprinting of lignin by ambient ionization high resolution mass spectrometry and simplified data mining. Analytica Chimica Acta 994:38–48 29126467 10.1016/j.aca.2017.09.012

[B8] Delcorte A., Garrison B.J., and Hamraoui K. (2009) Dynamics of molecular impacts on soft materials: from fullerenes to organic nanodrops. Anal Chem 81:6676–6686 20337378 10.1021/ac900746x

[B9] DeLeeuw J.W., and Largeau C. (1993) A review of macromolecular organic compounds that comprise living organisms and their role in kerogen, coal and petroleum formation. In Organic Geochemistry, edited by Engel M.H. and Mackos SA, Plenum Press, New York, pp 23–72

[B10] Everdell M.H. (1967) Some remarks on the thermodynamics of the xylenes. J Chem Educ 44:538

[B11] Glein C.R., Baross J.A., and Waite J.H. Jr. (2015) The pH of Enceladus' ocean. Geochim Cosmochim Acta 162:202–219

[B12] Goodacre R., and Kell D.B. (1996) Pyrolysis mass spectrometry and its applications in biotechnology. Curr Opin Biotechnol 7:20–28 8791308 10.1016/s0958-1669(96)80090-5

[B13] Goodfellow M., Freeman R., and Sisson P.R. (1997) Curie-point pyrolysis mass spectrometry as a tool in clinical microbiology. Zentralbl Bakteriol 285:133–156 9060148 10.1016/s0934-8840(97)80023-0

[B14] Gordon P.R., and Sephton M.A. (2016) Organic matter detection on Mars by pyrolysis-FTIR: An analysis of sensitivity and mineral matrix effects. Astrobiology 16:831–845. DOI: 10.1089/ast.2016.1485 27870586 10.1089/ast.2016.1485 PMC5124741

[B15] Greenberg R., and Geissler P. (2002) Europa's dynamic icy crust. Meteorit Planet Sci 37:1685–1710

[B16] Hall D.T., Strobel D.F., Feldman P.D., McGrath M.A., and Weaver H.A. (1995) Detection of an oxygen atmosphere on Jupiter's moon Europa. Nature 373:677–679 7854447 10.1038/373677a0

[B17] Hansen C.J., Esposito L., Stewart A.I., Colwell J., Hendrix A., Pryor W., Shemansky D., and West R. (2006) Enceladus' water vapor plume. Science 311:1422–1425 16527971 10.1126/science.1121254

[B18] Hartgers W.A., Sinninghe Damsté J.S., and de Leeuw J.W. (1994) Geochemical significance of alkylbenzene distribution in flash pyrolysates of kerogens, coals and asphaltenes. Geochim Cosmochim Acta 58:1759–1775

[B19] Hsu H.-W., Postberg F., Sekine Y., Shibuya T., Kempf S., Horanyi M., Juhasz A., Altobelli N., Suzuki K., Masaki Y., Kuwatani T., Tachibana S., Sirono S.I., Moragas-Klostermeyer G., and Srama R. (2015) Ongoing hydrothermal activities within Enceladus. Nature 519:207–210 25762281 10.1038/nature14262

[B20] Izumi A., andKuroda K.-i., (1997) Pyrolysis-mass spectrometry analysis of dehydrogenation lignin polymers with various syringyl/guaiacyl ratios. Rapid Commun Mass Spectrom 11:1709–1715

[B21] Jaramillo-Botero A., An Q., Cheng M.-J., Goddard W.A., Beegle L.W., and Hodyss R. (2012) Hypervelocity impact effect of molecules from Enceladus' plume and Titan's upper atmosphere on NASA's Cassini spectrometer from reactive dynamics simulation. Phys Rev Lett 109:213201 23215593 10.1103/PhysRevLett.109.213201

[B22] Johnson R.E., Burger M.H., Cassidy T.A., Leblanc F., Marconi M., Smyth W.H., McKinnon W.B., and Khurana K.K. (2009) Composition and detection of Europa's sputter-induced atmosphere. In Europa, edited by Pappalardo R.T., McKinnon W.B., Khurana K.K.; with the assistance of René Dotson with 85 collaborating authors, University of Arizona Press, Tucson, 507

[B23] Khurana K.K., Kivelson M.G., Stevenson D.J., Schubert G., Russell C.T., Walker R.J., and Polanskey C. (1998) Induced magnetic fields as evidence for subsurface oceans in Europa and Callisto. Nature 395:777–780 9796812 10.1038/27394

[B24] Killops S.D., and Killops V.J. (2005) Introduction to Organic Geochemistry. Blackwell Publishing, Oxford

[B25] Kivelson M.G., Khurana K.K., Russell C.T., Volwerk M., Walker R.J., and Zimmer C. (2000) Galileo magnetometer measurements: a stronger case for a subsurface ocean at Europa. Science 289:1340–1343 10958778 10.1126/science.289.5483.1340

[B26] Kivelson M.G., Khurana K.K., and Volwerk M. (2002) The permanent and inductive magnetic moments of Ganymede. Icarus 157:507–522

[B27] Lucejko J.J., Modugno F., Ribechini E., and del Rio J.C. (2009) Characterisation of archaeological waterlogged wood by pyrolytic and mass spectrometric techniques. Anal Chim Acta 654:26–34 19850164 10.1016/j.aca.2009.07.007

[B28] Lunine J.I. (2017) Ocean worlds exploration. Acta Astronaut 131:123–130

[B29] Matthewman R., Martins Z., and Sephton M.A. (2013) Type IV kerogens as analogues for organic macromolecular materials in aqueously altered carbonaceous chondrites. Astrobiology 13:9 10.1089/ast.2012.0820 23551239

[B30] Meuzelaar H.L.C., Haverkamp J., and Hileman F.D. (1982) Pyrolysis Mass Spectrometry of Recent and Fossil Biomaterials, Elsevier, Amsterdam

[B31] Pearson V.K., Sephton M.A., Franchi I.A., Gibson J.M., and Gilmour I. (2006) Carbon and nitrogen in carbonaceous chondrites: elemental abundances and stable isotopic compositions. Meteorit Planet Sci 41:1899–1918

[B32] Peters K.E., Walters C.C., and Moldowan J.M. (2005) The Biomarker Guide, Volume 2 Biomarkers and isotopes in Petroleum Exploration and Earth History. Cambridge University Press, Cambridge

[B33] Peuravuori J., and Pihlaja K. (1997) Pyrolysis electron impact mass spectrometry in studying aquatic humic substances. Anal Chim Acta 350:241–247

[B34] Phillips C.B., and Pappalardo R.T. (2014) Europa clipper mission concept: exploring Jupiter's ocean moon. Eos Trans AGU 95:165–167

[B35] Porco C.C., Helfenstein P., Thomas P.C., Ingersoll A.P., Wisdom J., West R., Neukum G., Denk T., Wagner R., Roatsch T., Kieffer S., Turtle E., McEwen A., Johnson T.V., Rathbun J., Veverka J., Wilson D., Perry J., Spitale J., Brahic A., Burns J.A., Delgenio A.D., Dones L., Murray C.D., and Squyres S. (2006) Cassini observes the active south pole of Enceladus. Science 311:1393–1401 16527964 10.1126/science.1123013

[B36] Postberg F., Kempf S., Hillier J.K., Srama R., Green S.F., McBride N., and Grün E. (2008) The E-ring in the vicinity of Enceladus: II. Probing the moon's interior—the composition of E-ring particles. Icarus 193:438–454

[B37] Postberg F., Khawaja N., Abel B., Choblet G., Glein C.R., Gudipati M.S., Henderson B.L., Hsu H.-W., Kempf S., Klenner F., *et al.* (2018) Macromolecular organic compounds from the depths of Enceladus. Nature 558:564–568 29950623 10.1038/s41586-018-0246-4 PMC6027964

[B38] Rakov V.S., Denisov E.V., Laskin J., and Futrell J.H. (2002) Surface-induced dissociation of the benzene molecular cation in Fourier transform ion cyclotron resonance mass spectrometry. J Phys Chem A 106:2781–2788

[B39] Roth L., Saur J., Retherford K.D., Strobel D.F., Feldman P.D., McGrath M.A., and Nimmo F. (2014) Transient water vapor at Europa's south pole. Science 343:171–174 24336567 10.1126/science.1247051

[B40] Sephton M.A. (2013) Aromatic units from the macromolecular material in meteorites: molecular probes of cosmic environments. Geochim Cosmochim Acta 107:231–241

[B41] Sephton M.A., and Botta O. (2005) Recognizing life in the Solar System: guidance from meteoritic organic matter. Int J Astrobiol 4:269–276

[B42] Sephton M.A., Looy C.V., Veefkind R.J., Visscher H., Brinkhuis H., and de Leeuw J.W. (1999) Cyclic diaryl ethers in a Late Permian sediment. Org Geoch 30:267–273

[B43] Sephton M.A., Verchovsky A.B., Bland P.A., Gilmour I., Grady M.M., and Wright I.P. (2003) Investigating the variations in carbon and nitrogen isotopes in carbonaceous chondrites. Geochim Cosmochim Acta 67:2093–2108

[B44] Sephton M.A., Verchovksy A.B., and Wright I.P. (2004) Carbon and nitrogen isotope ratios in meteoritic organic matter; indicators of alteration processes on the parent asteroid. Int J Astrobiol 3:221–228

[B45] Smith B.A., Soderblom L.A., Beebe R., Boyce J., Briggs G., Carr M., Collins S.A., Cook A.F. 2nd, Danielson G.E., Davies M.E., Hunt G.E., Ingersoll A., Johnson T.V., Masursky H., McCauley J., Morrison D., Owen T., Sagan C., Shoemaker E.M., Strom R., Suomi V.E., and Veverka J. (1979) The Galilean satellites and Jupiter: Voyager 2 imaging science results. Science 206:927–950 17733910 10.1126/science.206.4421.927

[B46] Sparks W.B., Hand K.P., McGrath M.A., Bergeron E., Cracraft M., and Deustua S.E. (2016) Probing for evidence of plumes on Europa with HST/STIS. Astrophys J 829:121

[B47] Sparks W.B., Schmidt B.E., McGrath M.A., Hand K.P., Spencer J.R., Cracraft M., and Deustua S.E. (2017) Active cryovolcanism on Europa? Astrophys J Lett 839:L18

[B48] Spencer J.R., and Nimmo F. (2013) Enceladus: an active ice world in the Saturn system. Annu Rev Earth Planet Sci 41:693–717

[B49] Spencer J.R., Tamppari L.K., Martin T.Z., and Travis L.D. (1999) Temperatures on Europa from Galileo photopolarimeter-radiometer: nighttime thermal anomalies. Science 284:1514–1516 10348736 10.1126/science.284.5419.1514

[B50] Summons R.E., and Powell T.G. (1987) Identification of aryl isoprenoids in source rocks and crude oils: biological markers for the green sulphur bacteria. Geochim Cosmochim Acta 51:557–566

[B51] Vékey K. (1996) Internal energy effects in mass spectrometry. J Mass Spectrom 31:445–463

[B52] Waite J.H., Glein C.R., Perryman R.S., Teolis B.D., Magee B.A., Miller G., Grimes J., Perry M.E., Miller K.E., Bouquet A., Lunine J.I., Brockwell T., and Bolton S.J. (2017) Cassini finds molecular hydrogen in the Enceladus plume: evidence for hydrothermal processes. Science 356:155–159 28408597 10.1126/science.aai8703

[B53] Woese C.R., Kandler O., and Wheelis M.L. (1990) Towards a natural system of organisms: proposal for the domains Archaea, Bacteria, and Eucarya. Proc Natl Acad Sci 87:4576–4579 2112744 10.1073/pnas.87.12.4576 PMC54159

[B54] Wörgötter R., Mair C., Fiegele T., Grill V., Märk T.D., and Schwarz H. (1997) Characterization of hydrocarbon cluster ion products by surface induced reactions. Int J Mass Spectrom Ion Processes 164:L1–L6

